# Desiccation survival in an Antarctic nematode: molecular analysis using expressed sequenced tags

**DOI:** 10.1186/1471-2164-10-69

**Published:** 2009-02-09

**Authors:** Bishwo N Adhikari, Diana H Wall, Byron J Adams

**Affiliations:** 1 Department of Microbiology and Molecular Biology, Brigham Young University, Provo, UT, USA; 2 Department of Biology and Natural Resource Ecology Laboratory, Colorado State University, Fort Collins, CO, USA; 3 Department of Biology and Evolutionary Ecology Laboratories, Brigham Young University, Provo, UT, USA

## Abstract

**Background:**

Nematodes are the dominant soil animals in Antarctic Dry Valleys and are capable of surviving desiccation and freezing in an anhydrobiotic state. Genes induced by desiccation stress have been successfully enumerated in nematodes; however we have little knowledge of gene regulation by Antarctic nematodes which can survive multiple environmental stresses. To address this problem we investigated the genetic responses of a nematode species, *Plectus murrayi*, that is capable of tolerating Antarctic environmental extremes, in particular desiccation and freezing. In this study, we provide the first insight into the desiccation induced transcriptome of an Antarctic nematode through cDNA library construction and suppressive subtractive hybridization.

**Results:**

We obtained 2,486 expressed sequence tags (ESTs) from 2,586 clones derived from the cDNA library of desiccated *P. murrayi*. The 2,486 ESTs formed 1,387 putative unique transcripts of which 523 (38%) had matches in the model-nematode *Caenorhabditis elegans*, 107 (7%) in nematodes other than *C. elegans*, 153 (11%) in non-nematode organisms and 605 (44%) had no significant match to any sequences in the current databases. The 1,387 unique transcripts were functionally classified by using Gene Ontology (GO) hierarchy and the Kyoto Encyclopedia of Genes and Genomes (KEGG) database. The results indicate that the transcriptome contains a group of transcripts from diverse functional areas. The subtractive library of desiccated nematodes showed 80 transcripts differentially expressed during desiccation stress, of which 28% were metabolism related, 19% were involved in environmental information processing, 28% involved in genetic information processing and 21% were novel transcripts. Expression profiling of 14 selected genes by quantitative Real-time PCR showed 9 genes significantly up-regulated, 3 down-regulated and 2 continuously expressed in response to desiccation.

**Conclusion:**

The establishment of a desiccation EST collection for *Plectus murrayi*, a useful model in assessing the structural, physiological, biochemical and genetic aspects of multiple stress tolerance, is an important step in understanding the genome level response of this nematode to desiccation stress. The type of transcript analysis performed in this study sets the foundation for more detailed functional and genome level analyses of the genes involved in desiccation tolerance in nematodes.

## Background

The Dry Valleys of Antarctica are one of the most extreme terrestrial environments on Earth [1]. Soils in this cold desert ecosystem are subjected to freezing temperatures, desiccation and salt accumulation that affect biological water availability [2,3]. Soil communities in Antarctic Dry Valleys are simple; primary production is largely limited to algae, and fauna are almost exclusively microbial grazers (mostly protozoa, rotifers, tardigrades and nematodes [4]). Nematodes are the dominant soil animals, present in 65% of the 415 soils sampled by Wall Freckman & Virginia [3] across four McMurdo Dry Valleys (MCM). Nematodes have been isolated from soil in an inactive coiled state called anhydrobiosis [5]. Anhydrobiosis is a survival strategy employed by nematodes, rotifers, and tardigrades in response to desiccation [6]. Nematodes in anhydrobiosis lose 95–99% of their body water content and can cease metabolic activity at any stage in their life cycle [7]. While in an anhydrobiotic state, nematodes are capable of surviving desiccation [8] as well as extreme cold [9]. Though Antarctic ecosystems are simple and have low species diversity compared to temperate ecosystems, nematodes are the most widely distributed and biologically diverse invertebrates in the Dry Valleys [5], with four species; *Scottnema lindsayae*, *Eudorylaimus antarcticus*, *Plectus antarcticus*, and *Geomonhystera antarcticola *[10]. It has been suggested that specimens identified as *P. antarcticus *de Man 1904 in MCM are *P. murrayi *[11,12] and we accept this nomenclature for the present paper.

*Plectus murrayi*, a bacteria feeding nematode [13], inhabits both semi-aquatic and terrestrial biotopes in the Dry Valleys, but is also reported from other parts of the Antarctica [11]. Similar to *S. lindsaye*, another nematode endemic to the Southern continent [14], *P. murrayi *has a multiple year life cycle [15]. The distribution of these nematodes in Antarctica is dependent on organic carbon and soil moisture [16] with high abundance in stream sediments [5]. *P. murrayi *from the MCM are freeze tolerant, and can tolerate repeated freeze-thaw cycles in the laboratory (data not shown). Although adapted to the extreme desiccation and freezing encountered in its habitat [12], the biology and environmental tolerance of this nematode has not been well studied.

Despite recent work on behavioral, biochemical and molecular stress response mechanisms [17-19] the molecular mechanisms governing anhydrobiosis in nematodes are not fully understood. Anhydrobiosis in nematodes is reported to involve the biosynthesis of low molecular weight carbohydrates, proteins and glycerol [20,21]. Recent research suggests anhydrobiotes synthesize many other compounds (primarily proteins) that are essential for survival [22-24]. Studies on these desiccation responsive compounds have resulted in the identification of many genes that play important roles in stress acclimation and survival. These responses include up-regulation of transcriptional regulators, molecular chaperones, antioxidants, hydrophilic proteins, and proteins involved in cell cycle regulation [19,25-27]. The anhydrobiotic nematode *Aphelenchus avenae *synthesizes large amounts of trehalose in response to desiccation [28]. However, it has become clear that such sugars are not sufficient for anhydrobiosis [29] and, indeed, that some anhydrobiotic organisms seem not to use them [30]. As an effort to identify other adaptations required for anhydrobiosis Goyal *et al*. [31] characterised genes in the nematode *A. avenae *that requires a period of preconditioning to enter anhydrobiosis. During this preconditioning period, several genes, including trehalose synthase [31], hydrophilins (highly hydrophilic proteins), anhydrin, and a polypeptide, *Aav-LEA-1*, related to plant Group 3 late embryogenesis abundant (LEA) proteins were induced [23,19]. Although similar gene classes were found to be associated with desiccation stress in many nematodes, none of the ESTs or proteins detected in these studies were encoded by the same gene [26] and their expression level was quite variable [32]. To understand such molecular mechanisms activated during anhydrobiosis, a condition induced by slow dehydration, we identified gene expression patterns by gradually desiccating nematodes at relative humidity (RH) values reflective of the Antarctic environment.

To understand the mechanisms of desiccation survival we have initiated a genomic level analysis of gene expression during anhydrobiosis of *P. murrayi*. The first step in this process was to establish an EST collection that is representative of the desiccation induced transcripts and to identify the transcripts differentially expressed during desiccation stress. Here we present bioinformatics and molecular analysis of 2,486 ESTs from the gradually desiccated and anhydrobiotically induced nematode *P. murrayi *and 80 transcripts differentially expressed during the anhydrobiotic process. The bioinformatics approaches include EST cluster analyses, transcript abundancy estimations, and functional classifications based on Inter-Pro domains, Gene Ontology hierarchy, and KEGG biochemical classifications. The genetic information derived from *P. murrayi *informs the characterization of genes responding to desiccation stress, and is expected to further our understanding of the potential genetic determinants of desiccation tolerance in nematodes and perhaps other metazoans.

## Results

### Sequencing and assembly of ESTs

**Figure 1:**
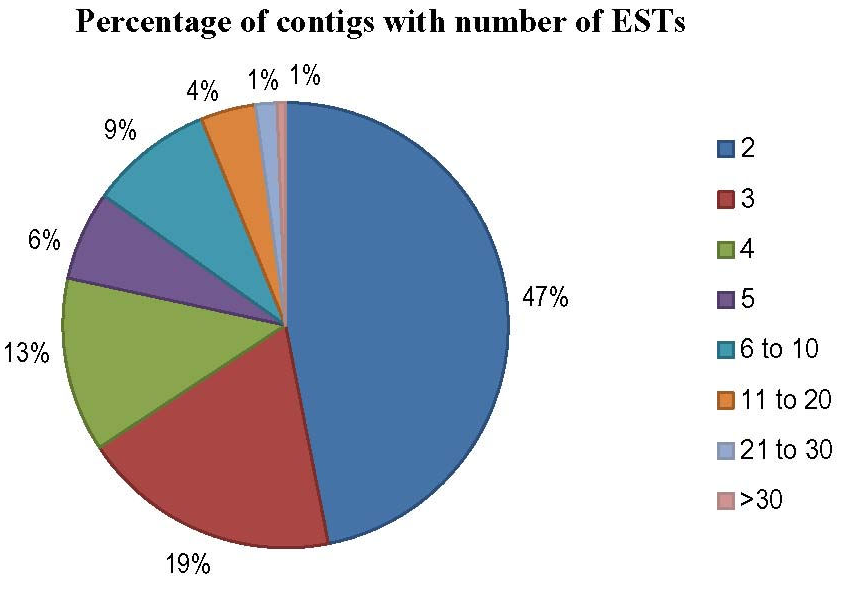
**Distribution of *Plectus murrayi *ESTs by cluster size**.

A directionally cloned cDNA library of desiccated nematodes was constructed and a total of 2,586 of clones were subjected to single pass sequencing from their 5' ends. Trimming of vector sequences, poly A/T tails, low quality, adaptor, and contaminating sequences provided a data set of 2,486 high quality (hq) ESTs with a minimum length of 100 base pairs (bp) (Table 1). Among 2,486 hq ESTs, 1,423 were assembled into a total of 324 contigs, and the remaining 1,063 ESTs were classified as singletons, suggesting a combined total of 1,387 putative unique transcripts (Table 1). The number of ESTs in the 324 contigs varied from 2 to 37, with the highest number of contigs being two ESTs, followed by more than 3 ESTs and the least number with more than 21 ESTs (Fig. 1). These hq ESTs ranged from 90–1125 bp with average lengths of 545 ± 156 bp. The average length of the contigs was higher than for singletons. The average GC content was higher in *P. murrayi *(44%) than in *C. elegans *(36%). All sequences have been deposited in the dbEST division of DDBJ/EMBL/GenBank under accession numbers [GenBank: FG618921] – [GenBank: FG621295], [GenBank: FG647736] – [GenBank: FG647869].

**Table 1:** *Plectus murrayi *EST summary

Total number of high quality sequences^‡^	2,486
Average length of sequences (bp)^†^	545 ± 156
Number of contigs^§^	324
Number of singletons	1,063
Number of putative unique transcripts^¶^	1,387
Unique transcripts with similarity to *C. elegans *database	523 (38%)*
Unique transcripts with similarity to other nematode database	106 (7%)
Unique transcripts with similarity to other organisms	153 (11%)
Total unique transcripts with significant similarity	782 (56%)
Unique transcripts with no significant similarity	605 (44%)

^‡^A sequence is considered high quality if it's trimmed PHRED 20 length is >100 bases after vector only, low-quality and contaminating sequences are removed.

^†^Calculated from the total ESTs.

^§^A contig (contiguous sequence) contains two or more ESTs.

^¶^Number of putative unique transcripts equals the number of contigs plus the number of singletons.

*Calculated as percentage of total unique transcripts with similarity at E < 10^-5^.

### Comparison against public nematode ESTs

We used the 1,387 unique sequences to search a non-redundant protein data base using BLASTX [33,34] (Table 1) and the Wormpep 190 database consisting of extensively curated *C. elegans *proteins from WormBase [35]. A total of 782 unique sequences (56%) matched known proteins, including 523 unique sequences (38%) with significant match to *C. elegans *proteins at a cut-off expectation (E)-value of 10^-5 ^or below. The remainder of the unique sequences (44%) had no meaningful matches (*E *> 10^-5^). We compared our unique sequences with the ESTs from other nematodes as well as non-nematodes using BLAST searches. Only 107 unique sequences (7%) matched other nematode ESTs and 153 unique sequences (11%) matched organisms other than nematodes at *E *< 10^-5 ^(Table 1). Of 1,387 unique sequences, 36 had homologues in *C. elegans *which could be silenced by RNAi. The RNAi phenotypes (as described by WormBase) included *mig-15 *(migration), *lin-8 *(lineage), *unc-16, 89 *(uncoordinated), *dpy-6 *(dumpy), *rde-1 *(RNAi defective), *drh-2 *(dicer related), *nhr-67 *(nuclear hormone receptor) and *ard-1 *(alcohol/ribitol dehydrogenase).

### Identification of differentially expressed genes

To identify transcripts differentially expressed (DE) during desiccation stress, subtractive hybridization was conducted between cDNA from gradually desiccated and fresh active nematodes (control). Two rounds of hybridization were done and DE clones were sequenced which resulted in 80 quality sequences above 100 bp (Table 2). The nucleotide sequences were analyzed and their putative functions identified by BLASTX search. The DE transcripts included 22 ESTs (28%) similar to metabolism related genes, 15 (19%) similar to environmental information processing genes, 23 (28%) similar to genetic information processing genes, 3 (4%) similar to hypothetical proteins of other organisms and 17 (21%) novel transcripts that had no identifiable similarity to known sequences in GenBank [36]. Among the metabolism related genes, 13 ESTs (68%) were involved in carbohydrate metabolism, 2 transcripts (10%) of each in lipid metabolism, amino acid metabolism and protein folding, sorting and degradation. The environmental information processing category was dominated (53%) by stress related proteins. In the genetic information processing category, ribosomal proteins were the most abundant (42%) group followed by translation elongation factor (19%) (Table 2). All sequences have been deposited in the dbEST division of DDBJ/EMBL/GenBank under accession numbers [GenBank: FK670236] – [GenBank: FK670315] and [GenBank: GH196899; GH229101].

**Table 2:** Listing of ESTs differentially expressed during desiccation of *Plectus murrayi *and their homologs in GenBank.

Clone ID	GenBank accession number	Homolog accession	Annotation (Organism) number	E-value	Percentage similarity
**Metabolism^ɠ ^(22)**					
SH_Pa.AA.01	FK670236	ref|NP_496237.1|	GPD family member [*Caenorhabditis elegans*]	1e-40	86%
SH_Pa.AB.02	FK670237	ref|NP_498081.2|	ALDH family member [*C. elegans*]	2e-80	74%
SH_Pa.AF.06	FK670241	gb|AAF81283.1|	Glutathione S-transferase [*Haemonchus contortus*]	3e-51	49%
SH_Pa.AH.08	FK670243	ref|NP_496161.1|	Lipid Transfer protein family member [*C. elegans*]	6e-42	72%
SH_Pa.AI.09	FK670244	gb|AAC47996.1|	Aspartyl protease protein 6 [*C. elegans*]	2e-51	53%
SH_Pa.EA.014	FK670249	WBGene00002263	Plant LEA related family member [*C. elegans*]	1e-33	28%
SH_Pa.FA.015	FK670250	gb|EDP32297.1|	TPS6 protein 1 [*Brugia malayi*]	1e-55	51%
SH_Pa.IA.018	FK670253	gb|EDP36623.1|	FBA1, putative [*B. malayi*]	1e-73	84%
SH_Pa.BD.021	FK670254	gb|AAC97508.1|	Thymidylate synthase [*C. elegans*]	1e-28	62%
SH_PA_Ssh.056	FK670287	WBGene00006975	Zinc finger protein family member [*C. elegans*]	2e-28	53%
SH_Pa.BH.025	FK670258	ref|NP_494721.1|	Probable glycerol kinase [*C. elegans*]	4e-62	71%
SH_Pa.BI.026	FK670259	ref|NP_001006395.1|	MDH1, NAD (soluble) [*Gallus gallus*]	1e-71	68%
SH_Pa.CC.027	FK670260	ref|ZP_00056387.1|	IDH [*Magnetospirillum magnetotacticum*]	4e-86	73%
PA_Sh_Ab.062	FK670293	ref|NP_498111.2|	ATP synthase subunit family member [*C. elegans*]	8e-110	93%
SH_Pa.CD.019	FK670261	gb|EDP37408.1|	NADH-ubiquinone oxidoreductase [*B. malayi*]	2e-57	60%
SH_Pa.CH.031	FK670264	ref|NP_496736.1|	Glycogen synthase family member [*C. elegans*]	1e-35	50%
SH_Pa.CI.032	FK670265	emb|CAA53718.1|	ADP/ATP translocase [*C. elegans*]	6e-93	90%
SH_Pa.DG.038	FK670269	ref|NP_503306.1|	Bi-functional glyoxylate cycle protein [*C. elegans*]	4e-95	90%
SH_Pa.DH.039	FK670270	gb|AAC19750.1|	Putative glutamate dehydrogenase [*H. contortus*]	9e-111	88%
SH_Pa.DH.030	FK670263	gb|AAC97508.1|	Thymidylate synthase [*C. elegans*]	1e-26	49%
SH_Pa.AA.036	FK670268	WBGene00009165	Glutathione peroxidase [*C. elegans*]	3e-63	79%
PA_Sh_bb.067	FK670298	ref|NP_498111.2|	ATP synthase sub unit family member [*C. elegans*]	8e-110	93%
					
**Environmental information processing (15)**					
SH_Pa.AC.03	FK670238	gb|AAM55195.1|	Cathepsin L cysteine protease [*H. contortus*]	2e-81	77%
SH_Pa.AD.04	FK670239	ref|NP_508913.1|	JNK kinase family member jkk-1 [*C. elegans*]	2e-06	41%
SH_Pa.AE.05	FK670240	WBGene00004930	Superoxide dismutase family member [*C.elegans*]	4e-80	81%
SH_Pa.AG.07	FK670242	gb|ABA41369.1|	Type II antifreeze protein [*Clupea harengus*]	2e-35	69%
SH_Pa.AA.010	FK670245	gb|AAN78300.1|	Heat shock protein 70 A [*Heterodera glycines*]	2e-59	89%
SH_Pa.DA.013	FK670248	ref|NP_495536.1|	Small heat shock protein family member [*C. elegans*]	2e-115	90%
SH_Pa.GA.016	FK670251	ref|NP_496549.1|	RAB family member [*C. elegans*]	7e-103	88%
SH_Pa.HA.017	FK670252	gb|EDP28446.1|	Ras-related protein Rab-11B, putative [*B. malayi*]	1e-53	77%
SH_Pa.BF.023	FK670256	ref|NP_509019.1|	Heat shock protein family member [*C. elegans*]	8e-78	91%
SH_Pa.BG.024	FK670257	gb|AAD00182.1|	Inhibitor of apoptosis homolog [*C. elegans*]	5e-53	52%
SH_Pa.CF.029	FK670262	gb|EDP35652.1|	Heat shock protein 90 protein [*B. malayi*]	8e-59	53%
SH_Pa.DD.034	FK670266	ref|NP_499889.2|	DumPY: shorter than wild-type [*C.elegans*]	7e-47	63%
PA_Sh_Ab.063	FK670294	gb|AAO44907.1|	Collagen protein 170 [*C. elegans*]	4e-30	62%
PA_Sh_Ib.070	FK670300	gb|EDP30373.1|	Leucine rich repeat family protein [*B. malayi*]	3e-27	40%
PA_Sh_bB.072	FK670302	gb|EDP31428.1|	Laminin receptor 1 [*Xenopus laevis*]	1e-73	71%
					
**Genetic information processing (23)**					
SH_Pa.BA.011	FK670246	gb|AAG50205.1|	AP inhibitor [*Parelaphostrongylus tenuis*]	7e-41	62%
SH_Pa.CA.012	FK670247	emb|CAJ57642.1|	Putative E2 enzyme [*Oesophagostomum dentatum*]	2e-74	97%
SH_Pa.DE.035	FK670267	gb|EDP39185.1|	Histone H2B 2, putative [*B. malayi*]	3e-33	94%
PA_Sh_Cb.064	FK670295	gb|AAT28331.1|	Peroxiredoxin [*H. contortus*]	5e-89	83%
PA_Sh_bH.078	FK670308	ref|NP_956267.1|	Ubiquitin specific protease 14 [*D. rerio*]	5e-40	44%
SH_Pa.DI.040	FK670271	ref|NP_491416.1|	Ribosomal protein, LSU family member [*C. elegans*]	7e-104	85%
SH_Pa.EE.041	FK670272	ref|NP_502794.1|	Ribosomal protein, SSU family member [*C. elegans*]	8e-73	94%
SH_Pa.EF.042	FK670273	ref|NP_502794.1|	Ribosomal protein, SSU family member [*C. elegans*]	3e-67	81%
SH_Pa.EG.043	FK670274	ref|NP_501167.1|	Ribosomal protein, SSU family member [*C. elegans*]	9e-85	84%
PA_Sh_EH.044	FK670275	ref|NP_741371.2|	Ribosomal protein, LSU family member [*C. elegans*]	5e-57	77%
PA_Sh_EI.045	FK670276	ref|NP_498660.1|	Ribosomal protein, LSU family member [*C. elegans*]	1e-67	88%
PA_Sh_Ab.046	FK670277	ref|NP_740944.1|	Ribosomal protein, SSU family member [*C. elegans*]	2e-40	82%
PA_Sh_Bb.047	FK670278	ref|NP_496375.1|	Ribosomal protein, LSU family member [*C. elegans*]	9e-51	97%
PA_Sh_Cb.048	FK670279	gb|EDP38710.1|	60S ribosomal protein L27a, putative [*B. malayi*]	5e-54	83%
PA_Sh_Db.049	FK670280	gb|EDP38220.1|	60S ribosomal protein L39, putative [*B. malayi*]	9e-21	94%
PA_Sh_Lb.057	FK670288	gb|EDP29175.1|	40S ribosomal protein S6, putative [*B. malayi*]	1e-25	86%
PA_Sh_Eb.050	FK670281	ref|NP_492457.1|	EF family member [*C. elegans*]	2e-66	89%
PA_Sh_Fb.051	FK670282	gb|EDP34276.1|	EF1-alpha, putative [*B. malayi*]	1e-117	88%
PA_Sh_Gb.052	FK670283	ref|NP_492457.1|	EF family member [*C. elegans*]	2e-66	89%
PA_Sh_Hb.053	FK670284	ref|NP_524808.2|	EF1 beta, isoform A [*Drosophila melanogaster*]	5e-45	68%
PA_Sh_Ib.054	FK670285	ref|NP_498520.1|	EF family member [*C. elegans*]	4e-100	89%
SH_Pa.BE.022	FK670255	WBGene00003623	NHR family member [*C. elegans*]	2e-06	38%
PA_Sh_Mb.058	FK670289	gb|EDP33960.1|	Transcription factor, putative [*B. malayi*]	9e-16	87%
					
**Hypothetical proteins (3)**					
PA_Sh_Jb.055	FK670286	ref|XP_001666153.1|	Hypothetical protein [*C. briggsae*]	4e-41	78%
PA_Sh_Ob.060	FK670291	ref|XP_001676045.1|	Hypothetical protein [*C. briggsae*]	3e-32	55%
PA_Sh_bC.073	FK670303	ref|XP_001631386.1|	Predicted protein [*X. laevis*]	2e-34	42%
					
**Novel proteins (17)**					
PA_Sh_Nb.059	FK670290	n.a	Novel	n.a.	
PA_Sh_Pb.061	FK670292	n.a	Novel	n.a.	
PA_Sh_Db.065	FK670296	n.a	Novel	n.a.	
PA_Sh_Eb.066	FK670297	n.a	Novel	n.a.	
PA_Sh_Gb.068	FK670299	n.a	Novel	n.a.	
PA_Sh_bA.071	FK670301	n.a	Novel	n.a.	
PA_Sh_bD.074	FK670304	n.a	Novel	n.a.	
PA_Sh_bE.075	FK670305	n.a	Novel	n.a.	
PA_Sh_bF.076	FK670306	n.a	Novel	n.a.	
PA_Sh_bG.077	FK670307	n.a	Novel	n.a.	
PA_Sh_bI.079	FK670309	n.a	Novel	n.a.	
PA_Sh_bA.080	FK670310	n.a	Novel	n.a.	
PA_Sh_bC.028	FK670311	n.a	Novel	n.a.	
PA_Sh_bD.020	FK670312	n.a	Novel	n.a.	
PA_Sh_bF.033	FK670313	n.a	Novel	n.a.	
PA_Sh_bG.037	FK670314	n.a	Novel	n.a.	
PA_Sh_bG.069	FK670315	n.a	Novel	n.a.	

^ɠ^Pathway assignment based on Kyoto Encyclopedia of Genes and Genomes (KEGG) classification.

The most unexpected discovery among DE ESTs was Type II antifreeze protein (AFP) [GenBank: FK670242 which showed high similarity to that of *Clupea harengus *(Table 2), the Atlantic herring. This finding represents another case of an ice structuring protein from an Antarctic nematode, suggesting the possibility that Antarctic nematodes may use similar antifreeze proteins for stress adaptation heretofore observed only in one Antarctic nematode [37], some fishes [38], insects [39], plants [40] and fungi and bacteria [41].

### Abundant transcripts expressed during desiccation

A total of 23 contigs containing 384 ESTs were highly redundant. This accounted for more than 15% of the total high quality ESTs. The minimum and maximum number of ESTs that made up these highly redundant contigs was 7 and 37 respectively (Table 3). More than one third (9) of the highly redundant contigs, totalling 127 ESTs, had significant similarity to various genes involved in metabolism. One third (8) of the highly redundant contigs, totalling 139 ESTs, had significant similarity to various environmental information processing related genes, indicating high transcript abundance of stress related genes, as expected. Two of the contigs, totalling 48 ESTs, had significant similarity to ribosomal proteins while three of the contigs, totalling 37 ESTs, had significant similarity to genetic information processing related genes. One of the highly redundant contigs totalling 33 ESTs matched similar sequences derived from the mitochondrial cytochrome oxidase subunit (Table 3). The most redundant group of contigs were composed of 37 ESTs and had significant similarity to ribosomal protein from *C. elegans*, indicating higher activities of ribosomal protein genes during desiccation stress.

**Table 3:** The most abundantly represented transcripts in the cDNA library

Contig No	Tentative annotation^§^	Number of ESTs	Percentage^₤ ^(%)
Contig_57	Ribosomal protein	37	1.48
Contig_132	Cytochrome c oxidase subunit 2	33	1.32
Contig_9	Small heat shock protein family	27	1.08
Contig_312	Rab-family member	25	1.00
Contig_87	Aquaporin	24	0.96
Contig_231	DNA-binding protein	23	0.92
Contig_32	Y-box family member	22	0.88
Contig_198	Zinc finger protein	18	0.72
Contig_54	Cu/Zn superoxide dismutase	17	0.68
Contig_56	Translation initiation factor	17	0.68
Contig_67	Chaperonin containing subunit	16	0.64
Contig_301	Glutamate synthase	13	0.52
Contig_287	NADH-dehydrogenase subunit 1	13	0.52
Contig_93	Cathespin b -like cysteine proteinase	13	0.52
Contig_74	Elongation factor 1 alpha	13	0.52
Contig_63	Glutathione S-transferase	12	0.48
Contig_152	Heat shock 70 kda protein	11	0.44
Contig_242	60S ribosomal protein	11	0.44
Contig_131	Elongation factor family member	11	0.44
Contig_29	Aldehyde dehydrogenase	7	0.28
Contig_149	Heat shock 90 kda protein	7	0.28
Contig_313	ATP synthase subunit	7	0.28
Contig_112	Nuclear hormone protein family	7	0.28

^§^Annotation based on most significant BLAST alignment for each cluster.

^₤^Percentage based on total number of high quality sequences.

### Functional classification based on gene ontology assignments

**Figure 2:**
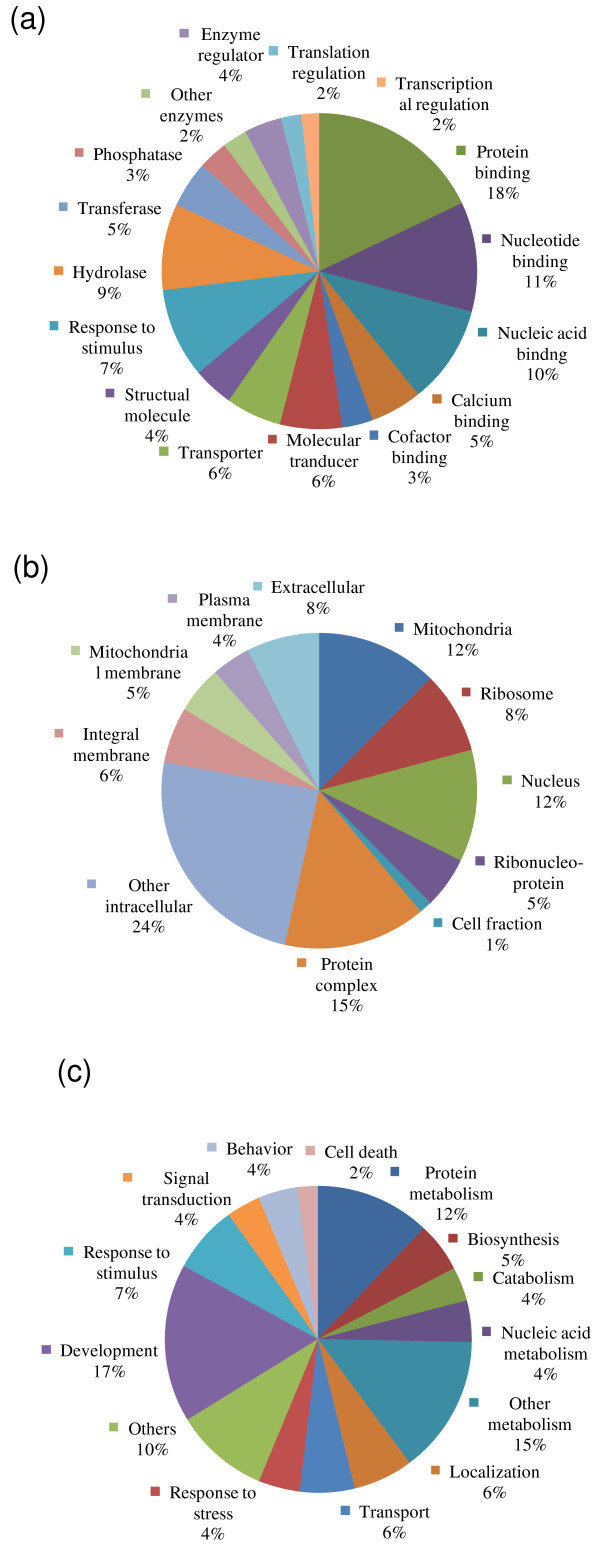
**Percentage representation of gene ontology (GO) mappings for *Plectus murrayi *clusters**. (A) Molecular functions; (B) Cellular components; and (C) Biological process. More detailed information is provided in Additional files 2a, 2b and 2c. Note that individual GO categories can have multiple mappings.

To categorize transcripts by putative function, we utilized the GO classification scheme (April 2008 release of GO database, Gene Ontology Consortium). GO provides a dynamic controlled vocabulary and hierarchy that unifies descriptions of biological, cellular and molecular functions across genomes [42]. In this report, we relied on well-annotated GO information of *C. elegans *and other nematodes. GO representation of *P. murrayi *clusters is shown for each organizing principle of GO: molecular functions (Additional file 1a; Fig. 1a), cellular components (Additional file 1b; Fig. 1b), and biological processes (Additional file 1c; Fig. 1c). Additional file 1 and Fig. 2 provide a breakdown of representation by major GO categories. The highest GO term for molecular functions was protein binding, under 'ligand binding and carrier' categories, which had 87 unique sequences accounting for 18% of the total unique sequences matched in this category and 6% of the total unique sequences. The highest final GO term in cellular components was mitochondria (under the 'cytoplasm' category) with a total of 28 unique sequences, 17 of which are encoded on the mitochondrial genome, accounting for 12% of the total in this category. Similarly, the highest final GO term for biological processes was protein metabolism, under 'metabolism' categories, which had 44 unique sequences accounting for 12% of the total in this category and more than 3% of the total unique sequences. We found 13 unique sequences showing significant similarity to *C. elegans *signal transduction factors; 8 of them belonged to the receptor binding group and 5 sequences belonged to receptor and receptor signalling proteins (Additional file 1).

### Functional classification based on KEGG analysis

As an alternative method of categorizing unique sequences by biochemical functions, sequences were assigned to metabolic pathways via KEGG [43] using enzyme commission (EC) numbers as the basis for assignment. Only 281 unique sequences (36% of total) were assigned EC numbers and had 158 unique mappings to KEGG biochemical pathways (Table 4). The KEGG metabolic pathways that are well represented by *P. murrayi *unique sequences are carbohydrate metabolism (18 enzymes), amino acid metabolism (9 enzymes), lipid metabolism (8 enzymes), xenobiotic and bio-degradation metabolism (5 enzymes), and biosynthesis of secondary metabolites (3 enzymes). Of these, 12% of the unique sequences belonged to the environmental information processing (EIP) category, indicating higher activities of stress and chaperone related genes during desiccation. The KEGG pathways well-represented under EIP are membrane transport (15 enzymes), ligand-receptor interaction (15 enzymes), signal transduction (8 enzymes) and signalling molecules and interaction (9 enzymes). About 11% of the unique sequences belonged to the genetic information processing (GIP) category with most of them having roles in folding, sorting and degradation. The KEGG pathways well-represented under GIP are folding, sorting and degradation (25 enzymes), transcription (9 enzymes), translation (8 enzymes) and replication and repair (6 enzymes). Most of the sequences (49%) remained unassigned to any known functional pathway and 15% of the sequences were similar to *C. elegans *hypothetical proteins (Table 4). The lowest number of sequences mapped to the cellular processes category (3%), suggestive of developmental arrest during anhydrobiosis. The cell growth and death (5 enzymes) and cell communication (4 enzymes) pathways were also the well-represented categories under cellular processes (Table 4).

**Table 4:** KEGG biochemical mappings for *Plectus murrayi *clusters.

KEGG categories represented	Unique sequences (Number of enzymes)	Percentage^§^
**Metabolism**	**84 (52)**	**11%**
Carbohydrate metabolism	29(18)	4%
Amino acid metabolism	14(9)	2%
Lipid metabolism	13(8)	2%
Xenobiotics biodegradation and metabolism	8(5)	1%
Biosynthesis of secondary metabolites	6(3)	<1%
Energy metabolism	5(2)	<1%
Nucleotide metabolism	3(2)	<1%
Metabolism of other amino acids	3(2)	<1%
Glyoxylate and dicarboxylate metabolism	3(3)	<1%
**Genetic information processing**	**83 (48)**	**11%**
Folding, sorting and degradation	42(25)	6%
Transcription	17(9)	2%
Translation	16(8)	2%
Replication and repair	8(6)	1%
**Environmental information processing**	**95 (47)**	**12%**
Membrane transport	30(15)	4%
Ligand-receptor interaction	28(15)	4%
Signal transduction	14(8)	2%
Signalling molecules and interaction	13(9)	2%
**Cellular processes**	**19 (11)**	**3%**
Cell growth and death	8(5)	1%
Cell communication	6(4)	<1%
Cell motility	3(1)	<1%
Development	2(1)	<1%
**Unassigned^‡^**	**385**	**49%**
**Hypothetical**	**116**	**15%**

^§^Percentage based on total unique transcripts (782) with significant similarity to sequences in database.

^‡^Unassigned sequences are those that have significant similarity to known sequences whose functions are unclear.

### Refined gene-specific expression using quantitative real-time PCR

**Figure 3:**
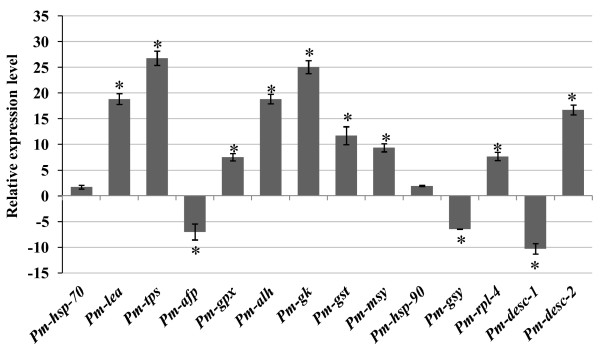
**Quantitative Real-time PCR analysis of gene expression in *Plectus murrayi *in response to desiccation**. Values were determined using qRT-PCR and represents relative expression of genes from desiccated to undesiccated nematodes (control). The relative expression of the target gene (*Pm-alh*: aldehyde dehydrogenase; *Pm-tps*: trehalose-6-phosphate synthase; *Pm-gpx*: glutathione peroxidise; *Pm-afp*: antifreeze protein; *Pm-hsp-70*: heat shock protein 70; *Pm-lea*: late embryogenesis abundant protein; *Pm-gk*: glycerol kinase; *Pm-ms*: malate synthase; *Pm-gsy*: glycogen synthase; *Pm-hsp-90*: heat shock protein 90; *Pm-rpl-4*: ribosomal protein-4; *Pm-desc-1*: novel protein I; *Pm-desc-2*: novel protein II; *Pm-gst-1*: glutathione s-transferase 1,) normalized to *Pm-18s*:18S rRNA and relative to the expression of control, was calculated for each sample using the 2^-ΔΔCT ^method [87]. Gene expression was determined in each sample using three independent technical replicates. A transcript with relative abundance of one is equivalent to the abundance of 18S rRNA. Bars represent standard errors calculated from three replicates of each experiment. *Significant difference (*P *< 0.05) from control.

In order to validate our differential gene expression results and obtain more refined gene expression data, we designed gene-specific primers for 14 transcripts selected from Table 2 and analyzed their expression using quantitative real-time PCR (qRT-PCR) (Fig. 3). These genes were chosen to represent a variety of functional classifications. Among these 14 transcripts, 9 were significantly induced (fold-change > 2.0×, *P *value < 0.05) and 3 genes were reduced (fold-change > 2.0×, *P *value < 0.05) in response to desiccation. Significant desiccation-induced gene expression change ranged from -6.50-fold for antifreeze protein to 26.77-fold for trehalose-6-phosphate synthase. Heat shock protein 70 and 90 were also weakly induced (fold-change 1.7 and 1.94×), but lacked significant statistical support *P *> 0.05). Among the DE transcripts, putative homologs to trehalose 6-phosphate synthase protein showed highest induction (fold-change 26.77×, *P *value < 0.05) followed by the putative homolog to glycerol kinase (fold-change 25.04×, *P *value < 0.05). Interestingly, there was significant reduction and induction on the level of expression of two novel transcripts, [GenBank: FK670306 and FK670310] (fold-change -10.3× and 16.71× respectively, *P *< 0.05), suggestive of their possible roles in desiccation tolerance (Fig. 3). The mRNA copy number was calculated using an absolute quantification method [44]. There was no significant difference (*P *value < 0.05) in copy number of transcripts encoding Hsp70 and Hsp90 on desiccated and control nematodes (Additional file 2).

## Discussion

The expressed genome of *P. murrayi *showed that anhydrobiotic survival in nematodes involves a suite of genes from diverse functional areas, such as hormone signaling transduction, transcription regulation, ROS scavenging, reestablishment of homeostasis, molecular chaperoning and transcriptional regulation of ribosomal proteins and other genes. The 2,486 sequences comprised 1,387 putative unique sequences from *P. murrayi *and represent the first EST analysis from an Antarctic nematode. The number of ESTs analyzed in the study were relatively few; nonetheless, 44% of the unique transcripts (605) expressed by desiccated nematodes represent novel genes, which falls within the range reported from several other nematode EST analyses [45]. Comparison of ESTs presented in this study with ESTs from GenBank showed that 56% of the unique transcripts (1,387) isolated from *P. murrayi *have been previously isolated from other organisms, including the model organism *C. elegans *(Table 1). Subtractive hybridization of cDNAs from desiccated and undesiccated nematodes used in library construction was done to enrich for the rare transcripts that are differentially expressed (DE). A total of 80 DE transcripts were identified, some of which are involved in genetic information processing followed by metabolism, and presumably are involved in stress survival of nematodes (Table 2). The expression level of 14 transcripts DE during desiccation survival using qRT-PCR indicates that these genes were DE between desiccated and undesiccated nematodes. The results from subtractive hybridization coupled with qRT-PCR showed that we appropriately enriched for the DE transcripts (Fig. 3). Our results showed that expressed sequence tag (EST) analysis coupled with subtractive hybridization is a powerful method to identify the genes involved in nematode desiccation stress. EST analysis is a commonly-used approach to identify genes involved in specific biological functions, especially in organisms where genomic data are not available [46].

### Functional analysis of expressed genes

Gene ontology has been widely used to characterize gene function annotation and classification [42]. GO describes gene function using controlled vocabulary and hierarchy, including molecular function, biological processes, and cellular components (Fig. 2; Additional file 1). A large number of the unique sequences from our study mapped to molecular functions and ligand binding, enzymes, molecular transducer and transporter subcategories. Each one of these subcategories represents catalytic activities that could be argued are important for a cell to survive major metabolic perturbation during desiccation. The second most represented category was biological process, which includes unique sequences associated with cell growth or maintenance, development and cellular communications. Within the cell growth or maintenance category most unique sequences were associated with metabolism, localization, transport and response to stress. This distribution is not surprising for ESTs (unique sequences) derived from an organism undergoing metabolic changes such as desiccation. The subcategories under the least represented category, cellular process, also seem to reflect the nature of the cellular disturbances that result from desiccation. Under this category there is significant representation under intracellular and membrane subcategories. Within these subcategories representation is most significant in mitochondria, cytosol, ribosome, nucleus and ribonucleoprotein complex. The importance of protein synthesis and cellular homeostastasis during desiccation may explain the preponderance of unique sequences associated with mitochondrial and ribosomal structural components.

As an alternative method of categorizing clusters by biochemical function, clusters were assigned to metabolic pathways using the KEGG database [44] (Table 4). Only 36% of the unique sequences mapped to the currently known KEGG pathways with 158 unique mappings. The paucity of EC assignments limits this aspect of analysis but the mapping of unique sequences to the KEGG metabolic and other pathways still presents some useful perspectives on the metabolic, protein folding and degradation and cellular repair emphasis of desiccating cells. Most of the unique transcripts belonged to genetic information processing (GIP) with protein folding, sorting and degradation. Transcripts encoding a cathespin L-like protease [GenBank: FK670238] enzyme found in this category have long been recognized for their role in intracellular and extracellular protein degradation in a range of cellular processes. In *C. elegans *cathespin L-protease (*Ce-cpl*) has been demonstrated to play a critical role during embryogenesis, larval development and moulting [47]. Considering the putative homology of the transcript encoding P. murrayi cathespin L-protease (Pm-cpl) with C. elegans, it might have a similar role in nematode molting and thus its possible role during desiccation survival needs further investigation. 

Metabolism was the second most represented category and pathways well-represented by the *P. murrayi *clusters were carbohydrate metabolism, amino acid metabolism, lipid metabolism, biosynthesis of secondary metabolites, glyoxylate, and decarboxylation metabolism (Table 4). The lipid metabolism pathway in anhydrobiotes is one of the most active pathways as lipids are the main reservoir of energy and the most likely source of carbon for the synthesis of trehalose [21]. The GIP category included the pathways involved in protein folding, sorting and degradation, transcription, translation, replication and repair. Desiccation stress coupled with oxidative stress results in lipid and protein damage, leading to impaired cellular survival and functioning. Nematodes are reported to respond to these damages and repair them with increased expression of stress-protective genes and antioxidant enzymes [48]. The cellular processes category has the least number of transcripts. This finding is suggestive of developmental arrest during anhydrobiosis. Indeed, anhydrobiosis may be an exaptation that allows the individual to survive unfavourable conditions by staying in an arrested state of development and reproduction (Table 4).

### Abundantly expressed transcripts during anhydrobiosis

A high level of representation in a cDNA library generally correlates with high transcript abundance in the original biological sample [49], although artefacts of library construction can result in selection for or against representation of some transcripts. The genes with the most abundant transcripts were mostly involved in metabolism, molecular chaperones, reactive oxygen species scavenging and genetic information processing (Table 3). Ribosomal protein (RP) (GenBank: FG618944) was the most abundant transcript expressed during desiccation, suggesting the possible involvement of transcriptional regulation of ribosomal proteins during desiccation stress. Differential expression of plant ribosomal protein genes has been observed during development and following various stress or hormone treatments, including desiccation stress [50]. The 'ribosomal filter hypothesis' of Mauro and Edelman [51] proposes that particular combinations of ribosomal proteins or rRNA could favour the translation of specific mRNAs, thereby providing a mechanism for translational control. Interestingly, many ribosomal proteins were DE and one of them was up-regulated (*Pm-rpl-4*), suggesting that RPs may provide a means for selectively translating specific mRNAs required for the desiccation response as suggested for the insect parasitic nematode *Steinernema carpocapsae *[28]. Similarly, it may be part of an adaptive response to maintain ribosomal function in dehydrated cytoplasm.

A number of metabolism related transcripts encoding cytochrome c oxidase subunit 2 (GenBank: FG618989), glutamate synthase (GenBank: FG620419), NADH-dehydrogenase subunit 1 (GenBank: FG619004) and aldehyde dehydrogenase (GenBank: FG619103) were also abundantly expressed during desiccation stress. Several lines of evidence have suggested that nematodes activate metabolic pathways in response to desiccation shortly after exposure to dehydrating conditions [26].

Transcripts of a number of stress related genes were also abundantly expressed, including heat shock proteins, a chaperonin containing sub-unit, multiple stress-responsive zinc finger proteins and oxidative stress responsive genes. Desiccation stress in nematodes reportedly produces a large number of molecular chaperones to facilitate the synthesis, folding, assembly and intracellular transport of proteins, reduce protein denaturation and aggregation, and aid in protein renaturation [19,52]. Transcripts of several oxidative stress related genes like glutathione S-transferase (GenBank: FG618951) and copper/zinc superoxide dismutase (GenBank: FG618942) were also abundantly expressed. Reactive oxygen species and other toxins produced by oxidative stress during desiccation of nematodes can damage membrane systems, proteins and nucleic acids. Therefore, the transcript abundance of several proteins that contribute to cellular survival after oxidative damage is not surprising.

### Genes of general and secondary metabolism

Nematodes activate their metabolic pathways in response to desiccation shortly after exposure to dehydration [26]. Several transcripts encoding metabolism related genes are differentially expressed by desiccation stress in *P. murrayi*. These genes include aldehyde dehydrogenase [GenBank: FK670237], trehalose-6-phosphate synthase [GenBank: FK670250], thymidylate synthase [GenBank: FK670263], glycerol kinase [GenBank: FK670258], glycogen synthase [GenBank: FK670264], ATP synthase [GenBank: FK670293], ADP/ATP translocase [GenBank: FK670265], and malate dehydrogenase [GenBank: FK670259]. Interestingly, a transcript encoding a bifunctional glyoxylate cycle protein (malate synthase), a distinct and anaplerotic variant of the tricarboxylic acid cycle, was also found to be highly expressed during desiccation (Table 2).

Nematodes are unique among animals in utilizing the glyoxylate cycle to generate carbohydrates from the beta-oxidation of fatty acids [53]. The glyoxylate pathway, generally found in plants and micro-organisms, is similar to the citrate cycle, but relies on two critical enzymes, malate synthase and isocitrate lyase, to bypass two decarboxylation steps. Interestingly, the anhydrobiotic nematode *A. avenae *has been reported to use the glyoxylate cycle during induction of anhydrobiosis [54]. One sequence unique to *P. murrayi *that mapped to a glyoxylate cycle protein [GenBank: FK670269] includes putative homologs of malate synthase (*Pm-ms*). The abundant expression of malate synthase transcripts in our EST collection and its up-regulation upon desiccation stress may provide experimental support for an active role of glyoxylate cycle proteins during induction of anhydrobiosis by *P. murrayi*.

### Transcriptional regulation and signalling affected by desiccation

Transcriptional regulation and intracellular signalling cascades for nematode stress response in general and secondary metabolism in particular, are poorly understood. A number of desiccation responsive *P. murrayi *ESTs encode putative signalling molecules or transcription factors. One of the *P. murrayi *ESTs was most similar to the *unc-16 *gene of *C. elegans*, which encodes a c-Jun N-terminal kinase (JNK)-interacting protein [55], and one of the members of JNK kinase family (*jkk-1*) [GenBank: FK670239] was DE during desiccation. JNK (also known as stress-activated MAP kinases or SAPK) is a member of the mitogen-activated protein kinases (MAPKs) that regulate cellular responses to a variety of extracellular signals, including desiccation stress [56-58]. Transcripts homologous to genes encoding Zinc finger protein (GenBank: FG619080) were abundantly present in the expressed genome of *P. murrayi*. Zinc finger proteins are cellular proteins which play a major role in transcriptional regulation by binding with high affinity to specific regions of DNA. In conjunction with leucine zipper domains these proteins may form hetero- or homodimers and activate transcription, either constitutively, or in a regulatory manner, through post-translational modifications in response to external stimuli (although some may also be cell specific or developmentally regulated) [59]. The abundance of genes encoding zinc finger protein in response to desiccation suggests that these proteins may regulate further events in the stress-response cascade of *P. murrayi*. Three transcripts from *P. murrayi *were most similar to the *C. elegans *gene encoding a predicted neurotransmitter gated ion-channel (GenBank: FG620960) protein. Neuronal signal transduction in response to desiccation stress would be required to initiate the coiling response [60,70] of desiccating *P. murrayi*.

### Stress response genes expressed during anhydrobiosis

Nematodes respond to desiccation stress by synthesizing a conserved set of proteins [23-25]. Our results demonstrate that for *P. murrayi *desiccation stress can significantly elevate stress related genes encoding trehalose 6-phosphate synthase, late embryogenesis abundant proteins, heat shock proteins, ubiquitin, c-type lectins, chaperone related proteins, and other stress responsive genes (Table 3). Three transcripts encoding trehalose 6-phosphate synthase, which synthesize the storage carbohydrate trehalose, were expressed during desiccation stress. A characteristic feature of anhydrobiotic organisms is their synthesis of high concentrations of non-reducing sugars during the induction of anhydrobiosis [29,61]. Trehalose protects membranes and proteins from desiccation damage by replacing structural water [61], and contributes to the formation of an intracellular organic glass [62] which is thought to stabilize the cell's contents. The up-regulation of trehalose during desiccation stress of *P. murrayi *could be part of an adaptive response to desiccation (Fig. 3).

Protein aggregation during desiccation is likely to be a major potential hazard for anhydrobiotes; late embryogenesis abundant (LEA) proteins acting as molecular chaperones or molecular shields play an important role in prevention of this aggregation [63]. Transcripts similar to plant LEA related family members [GenBank: FK670249] of *C. elegans *were up-regulated during desiccation stress of *P. murrayi*. An LEA group 3 gene *Aav-lea-1 *was strongly induced in *A. avenae *during the induction of anhydrobiosis [24]. The *C. elegans *genome encodes three LEA genes [20] and silencing of the *lea-1 *gene by RNA interference (RNAi) caused a marked reduction in desiccation resistance in dauer larvae [64]. We thus assume that LEA proteins contribute to protection and recovery from desiccation stress in anhydrobiotic nematodes.

Molecular chaperones, such as the Hsp70 family and the Hsp60 chaperonin complexes, are commonly perceived as heat shock proteins (Hsps), being up-regulated by stress. The *P. murrayi *transcripts of 70 [GenBank: FK670245] and 90 kda heat shock protein [GenBank: FK670262] and small heat shock proteins were abundantly expressed following desiccation stress. Heat shock proteins have been implicated in response to desiccation in many nematodes [18], but they appear to be constitutively expressed in *P. murrayi *(Additional file 2). Though Hsps may contribute to enhanced stress resistance overall, our results showed no evidence that expression levels of these Hsps were altered by desiccation. It has been shown that other Antarctic organisms constitutively express Hsp*70 *and Hsp90, showing no or modest up-regulation of this gene in response to thermal stress [65-67]. Characteristically, Hsps are expressed not at the animal's normal habitat condition, but only as part of the organism's stress response. In fact, the expression of these genes is thought to be incompatible with ongoing protein synthesis and the progression of development [68,69]. It may be that *P. murrayi *has evolved a mechanism to maintain Hsp function without disrupting normal metabolism and growth that requires synthesis of other proteins. Consistent with the observed constitutive expression, it is possible that desiccation stress did not activate these genes, and the mild desiccation failed to boost Hsp expression. An alternative explanation for the continuous up-regulation of Hsps is that because Antarctic nematodes are frequently, although unpredictably, exposed to a variety of environmental stressors such as desiccation, high pH, extreme osmotic shock, freezing, and anoxia as well as temperature [70], their survival depends on maintaining continuous expression of molecular chaperones. Furthermore, because of the unpredictability and the potential rapidity of exposure to diverse environmental stresses, the continuous production of these molecular chaperones may be energetically justified.

Unexpectedly, one of the ESTs encoded a protein similar to the type II antifreeze protein (AFP) [GenBank: FK670242] of Atlantic herring (*Clupea harengus*). This finding is somewhat surprising since antifreeze proteins from phylogenetically divergent organisms (insects, fish, plants, etc.,) generally have very little similarity to each other [71]. The transcript encoding antifreeze protein (AFP) (*Pm-afp) *was down-regulated during desiccation stress of *P. murrayi*. It is found that *Pm-afp *is up-regulated only during freezing and once nematodes are exposed to desiccation other stress response mechanisms are activated by down-regulating AFP (B.N. Adhikari, unpublished work). Many overwintering organisms, including insects, fish, bacteria, fungi, and plants, accumulate AFPs that bind to the faces of ice crystals during freezing and inhibit their growth [71]. While the high degree of AFP similarity between *P. murrayi *and *C. harengus *raises the idea that it originated in either of the two organisms via horizontal gene transfer, we suggest that such speculation is premature, particularly given their geographic, ecological, and phylogenetic disparity, but also because such a small fraction of the phylogenetic diversity of nematode genomes has been explored. Further sequencing and characterization are currently underway to better understand the origin and evolution of this gene in *P. murrayi*.

### Oxidative stress genes expressed during anhydrobiosis

Desiccation stress induces the generation of reactive oxygen species (ROS) in nematodes, and therefore it is important for nematodes to have effective ROS-scavenging mechanisms. A number of ESTs encoding proteins which detoxify reactive oxygen species like superoxide dismutase (SOD) [GenBank: FK670240], Ras-related protein [GenBank: FK670252], and glutathione S-transferase (GST) (GenBank: FK670241) were expressed in *P. murrayi *in response to desiccation (Table 2). The SOD enzymes are a family of metalloenzymes responsible for quenching the potentially deleterious effects of superoxide radicals. There was abundant expression of ESTs similar to *C. elegans sod-1 *that encode a copper/zinc superoxide dismutase (Table 3). Three transcripts encoding glutathione s-transferase 1 were expressed in the ESTs of *P. murrayi *and one of them was up-regulated following desiccation stress. GSTs are a diverse super-family of multifunctional proteins that play prominent roles in detoxification metabolism in nematodes [72]. More than a dozen different GSTs have been isolated from *C. elegans *[73] and these detoxifying enzymes are reported to be involved in several functions, including xenobiotic detoxification and oxidative stress tolerance [72,74]. Differential expression and up-regulation of detoxifying enzymes like *Pm-sod-1 *and *Pm-gst-1 *suggests that *P. murrayi *has efficient ROS scavenging mechanisms under desiccation stress.

### Membrane and transport-related protein expressed during desiccation

In this study, we identified several ESTs encoding proteins involved in transport facilitation. A total of 15 genes encoding ion and water transporters included ABC transporter proteins (GenBank: GH229101), water channel proteins (GenBank: GH196899), ATPase (GenBank: FG618924) and lipid transfer proteins (LTP) [GenBank: FK670243]. Though the mechanism of coupling ion and water flow through membrane channels is not well studied, there is evidence that up-regulation of such genes is correlated with sensitivity to different types of stress in nematodes [75]. Sixty ABC transporters have been identified and functionally characterized in C. elegans. Members of this protein family are responsible for resistance to heavy metals [76]. In addition to short-term response and regulatory mechanisms, a functional system for reestablishing homeostasis is vital to desiccation tolerance. We found transcripts encoding proteins involved in ion homeostasis, such as vacuolar H+-ATPase and ATP synthase [GenBank: FK670298], showing that P. murrayi has efficient homeostatic pathways. Desiccation tolerance involves changes in the levels and composition of fatty acids of the major glycerolipids in nematodes [21]. Our data showed that gene encoding non-specific LTP was differentially regulated following desiccation, suggesting an active lipid metabolism and desiccation resistance.

### Refined gene-specific expression using quantitative real-time PCR

In general, we observed larger changes in gene expression using qRT-PCR, likely reflecting the greater dynamic range of detection and sensitivity of this method for gene expression profiling. Desiccation stress caused significant up-regulation of transcripts encoding stress related genes like *Pm-lea *(late embryogenesis abundant protein) and *Pm-tps *(trehalose-6-phosphate synthase) and down-regulation of *Pm-afp *(antifreeze protein). Similarly, there was significant up-regulation of the ROS scavenging enzyme *Pm-gst-1*(glutathione s-transferase 1) and metabolism related genes like *Pm-gpx *(glutathione peroxidise) and *Pm-ms *(malate synthase), and down-regulation of *Pm-afp *and *Pm-gsy *(glycogen synthase). Similar gene expression patterns, except for antifreeze protein, were reported for the free-living mycophagous nematode *A. avenae *[20] and insect parasitic nematode *Steinernema feltiae *[26] during desiccation acclimation and survival (Fig. 3). Although the observed changes in gene expression are common to many nematodes (except for the antifreeze protein and heat shock proteins), we observed significant variability in the magnitude of transcript abundance in *P. murrayi*. It is likely that the expression of these genes in response to desiccation stress is influenced by the thermal history of the organism, including seasonal variation experienced in the field, and by phylogenetic constraints.

## Conclusion

Nematodes have an important yet poorly understood place in the study of stress response survival, and desiccation tolerance in particular. Nematodes are the most abundant metazoans on earth [77] and prominent drivers of Antarctic ecosystem functioning [78]. Similar to the Antarctic nematode *Panagrolaimus davidi *[79], *P. murrayi *are desiccation as well as freeze tolerant, which establishes them as a useful model in assessing the structural, physiological, biochemical and genetic aspects of multiple stress tolerance, and the mechanisms by which organisms respond to and survive in extreme environments. To date very few studies have focused on the molecular aspects of desiccation tolerance in Antarctic nematodes and no genomic data is available in existing databases. The progression of Antarctic nematode genomics will be critical to the development of more useful and manipulable models for understanding desiccation tolerance and the nature of extremophiles. It is to these ends that we have initiated this study into the transcriptome of *P. murrayi *as they respond to a major stress event, in this case desiccation.

In the present study of 2,486 ESTs, a significant portion of transcripts had no known homologue in any nematode or other organisms for which sequence data are currently available in public databases. These molecules are particularly interesting as they may represent genes that are species/lineage specific or unique to Antarctic nematodes. There is considerable scope in exploring such molecules in the future and using a combination of genomic and proteomic approaches could provide valuable information on the survival strategy of extremophiles. Although the number of transcripts analyzed under this study is not large, we have identified and validated several genes differentially expressed during desiccation stress. It is crucial to emphasize that changes in mRNA accumulation may not necessarily correlate with protein/enzyme activity level [80]. However, the expression profiles provide starting points for more in-depth studies on candidate genes using additional genetic approaches. The annotation of transcripts with significant fold change and detection of consistently DE transcripts by SSH and qRT-PCR strongly suggest that these putative genes have an important role in *P. murrayi *desiccation stress response. In combination with the bioinformatics analysis presented here, further functional genomics analyses are required to generate a more complete picture of the cellular response of a free-living metazoan to extreme abiotic stress and determine the biological roles of these genes within the larger context of its Antarctic ecosystem.

## Methods

### Nematode culture

Live nematodes (*P. murrayi*) from Antarctica were reared in culture in order to produce sufficient quantities for experiments. Nematodes were cultured in sand agar plates containing Bold Modified Basal Freshwater Nutrient Media (BMBFN) (Sigma Aldrich Inc. USA) and stored at 15°C until used. Briefly, 20 ml/L BMBFN solution was mixed with 15 g/L Bacto-Agar in deionised water and autoclaved. The autoclaved Agar media was poured into Petri dishes and Standard Ottawa Sand (EMD Chemicals Inc., Gibbstown, NJ) was spread over each plate before the media solidified. About 5–10 nematodes were released into each plate, and a thin layer of water was maintained on the surface of the media for the duration of culture. The plates were incubated at 26°C for one to two weeks followed by 16°C for 3–5 weeks. In the laboratory culture at 16°C, nematodes completed their life cycle in about 6–8 weeks. Nematodes were also cultured in Nematode growth medium (NGM) plates with *Escherichia coli *OP50. About 15–20 nematodes were placed in one NGM plate and incubated at 16°C for about 5–8 weeks. Nematodes were sub-cultured every 3–4 weeks by chunking the agar with a sterile scalpel and moving a chunk of agar from an old plate to a fresh plate containing a lawn of *E. coli *OP50. To harvest the nematodes, the Agar media was cut into small pieces and poured into #40 standard testing sieve (Advantech Manufacturing, New Berlin, WI). Agar was removed by sieving and nematodes were washed twice with deionized water before collection by centrifugation.

### Desiccation treatment

Nematodes (about 3,000–5,000) were desiccated in glass desiccation chambers. The relative humidity (RH) was controlled by using saturated salt solutions as described by Winston & Bates [81] with some modifications. The requisite RH was maintained at 23°C ± 1°C in the desiccation chamber for 3 days prior to the addition of nematodes for equilibration. Relative humidity was maintained as 100% RH with distilled water vapour; 97% with K_2_SO_4_, 87% RH with HCl and 75% with NaCl. The treatments were an initial desiccation at 97% RH for 3 days followed by exposure to 87% for 2 days. All experiments were repeated under identical conditions using mixed stage populations of *P. murrayi*.

### RNA isolation

The desiccated and control nematodes were transferred to 10 volumes of Trizol Reagent (Molecular Research Center Inc., Cincinnati, OH) and exposed to freeze thaw cycles using liquid nitrogen and a 37°C water bath. The suspension was ground using mortar and pestle and vortexed. RNA was phase separated using chloroform, precipitated by isopropanol and pelleted. Total RNA was quantified and quality checked by spectrophotometer (NanoDrop, ND-1000 Spectrophotometer) and running agarose gel with RNA Century™ Plus Marker (Ambion Inc., Austin, TX).

### cDNA Library Construction and Sequencing

Total RNA was extracted from the gradually desiccated nematodes and used as starting material for complementary deoxyribonucleic acid (cDNA) library construction. A full-length cDNA library was prepared using Creator™ SMART™ cDNA library construction kit (Clontech, Mountain View, CA) according to the manufacturer's protocol. Briefly, 2 μg of total RNA was reverse transcribed to cDNA using SMART™ IV Oligonucleotide and PowerScript Reverse Transcriptase and amplified using long distance (LD) PCR. The cycling parameters were 95°C, 20 s; two 20 cycles of 95°C, 5 s; and 68°C, 6 min. cDNA was analyzed using agarose gel electrophoresis, proteinase K digested and restriction digested by *SfiI*. cDNA was size fractionated using CHROMA SPIN-400 columns and analyzed via agarose gel electrophoresis. The size fractionated cDNA was directionally cloned into pDNR-LIB vector and incubated at 16°C for 15 h. Transformation was done at 37°C overnight, and colonies were picked and grown for 18 h. Template DNA was extracted using QIAprep Spin Miniprep Kit (Qiagen Inc., Valencia, CA), amplified, cleaned using ExoSAP-IT^® ^(USB Corporation, Cleveland, Ohio) and sequenced using an ABI PRISM 377 automated DNA sequencer (Applied Biosystems, CA) at the DNA Sequencing Center (DNASC) at Brigham Young University (BYU). Sequencing reactions were performed with 2 μl of ABI PRISM BigDye Terminators v3.0 (Applied Biosystems) and 3.5 pmol of primer in 12 μl reaction volumes, followed by a sequencing reaction clean up to remove residual dye and enzyme. Sequencing was with M13 forward (GTAAAACGACGGCCAG) and reverse (CAGGAAACAGCTATGAC) primers for 25 cycles of 96°C, 10 s; 50°C, 5 s; and 60°C, 4 min. The sequenced products were sorted and analyzed with Sequencher™ 4.8 (Gene Codes Corporation, Ann Arbor, MI).

### Subtractive Hybridization

Subtractive hybridization was performed with the PCR-Select™ cDNA subtraction kit (Clontech, Palo Alto, CA). Random hexamer primers (Clontech) were used to convert mRNA that had been extracted from desiccated and control nematodes into cDNA. The cDNA under investigation was designated tester, and the reference cDNA was designated driver. In the forward run cDNA from desiccated samples was designated as tester, in a reverse run control nematode cDNA was used as tester to investigate both up- and down-regulation of mRNA molecules. Second strands of driver and tester DNA were synthesized using T4 DNA polymerase and restriction digested by *RsaI *to create blunt-ended double stranded cDNA for subtraction. The tester cDNAs were subdivided into two portions and each group ligated to different adaptor sequence and subjected to two levels of subtractive hybridizations as described in the manufacturer's protocol. The DE cDNAs were selectively amplified by two PCR reactions using 50× Advantage cDNA Polymerase mix. The first PCR amplified double stranded cDNA with different adaptor sequences and the second PCR further reduced background and enriched for DE sequences. The PCR-amplified cDNA fragments generated by SSH were then ligated into the TOPO^® ^TA Cloning^® ^vector (Invitrogen Corporation, USA) and sequenced using ABI 3730xl DNA Analyzer (Applied Biosystems, CA) at the BYU DNASC.

### Sequence analysis and EST clustering

Base calling was performed using PHRED software (versions 0.000925.c) [82,83] with the quality cut-off set at PHRED 20. Raw sequences were then imported into the Vector NTI Advance™ 10 software (Invitrogen Corporation) and subjected to trimming of vector sequences and 5' adapter sequences (for subtractive hybridization) using default settings. Sequences with less than 100 quality bases (PHRED 20 or better) after trimming were discarded. Sequences having polyA tails of 100 bases or more were eliminated from analysis. EST sequences representing contamination from bacterial, yeast or fungal sources were identified using BLASTN algorithm [34,35] and removed from further analyses. ESTs were aligned and assembled into contigs using CAP3 software [84] when the criterion of a minimum identity of 95% over 50 bp was met. When an EST could not be assembled with others in a contig, it remained as a "singleton". The contigs and the singletons should thus correspond to sequences of unique genes. The consensus sequences of the contigs and the sequences of the singlets were compared to the sequences in GenBank's non-redundant (nr) and Uniprot database using the TBLASTX and the BLASTX [38,39] algorithms and *C. elegans *Wormpep 190 database [36] using the BLASTX algorithm [35]. The cut-off for sequence similarity was E-value < 10^-5 ^for all analyses.

### Functional analysis and pathway assignments

Gene ontology (GO) term annotation and function-based analysis [85] of unique sequences were performed using Blast2go (V 1.6.2) [86], a sequence-based tool to assign GO terms, extracting them for each BLAST hit obtained by mapping to extant annotation associations. GO terms for each of the three main categories (biological process, molecular function, and cellular component) was obtained from sequence similarity using the application default parameters. From these annotations, pie charts were made using 2nd level GO terms based on biological process, molecular function, and cellular component. Pathway assignments were carried out according to Kyoto encyclopedia of genes and genomes (KEGG) mapping [43]. Enzyme commission (EC) numbers were assigned to unique sequences that had BLASTX scores with a cut-off value of *E *= 10^-5 ^or less upon searching protein databases. The sequences were mapped to KEGG biochemical pathways according to the EC distribution in the pathway database.

### Primer Design

All the primers used in quantitative real-time PCR (qRT-PCR) were designed with IDT SciTools (Integrated DNA Technologies, Coralville, IA) by aligning EST sequences with similar sequences from NCBI. All the primers used in this study were synthesized by Operon (Operon Biotechnologies Inc., Huntsville, AL). The sequences of the primers and product sizes are listed in Additional file 3.

### Quantitative Real-time PCR

Total RNA extracted from dehydrated and control *P. murrayi *nematodes was reverse transcribed using ImProm-II™ reverse transcriptase (Promega corporation, Madison, WI) and subjected to qRT-PCR analysis using Light cycler 480 SYBER Green I mastermix and gene specific primers in a Light cycler 480 RT-PCR system (Roche Applied Science, Mannheim, Germany) equipped with light cycler 480 software. High-resolution gel electrophoresis was used to verify that the qRT-PCR amplification product from each examined gene was a single-band product. Thermal cycling was performed in accordance with the manufacturer's instructions for a total of 40 cycles at an annealing temperature of 58°C for each primer pair. Quantitative RT-PCR analysis was performed with Lightcycler 480 software, the threshold cycle was automatically calculated by the second-derivative maximum method, and the copy number of the specific mRNA in the experimental samples was calculated by extrapolation from the gene-specific standard curve.

### Data analyses

The copy number of specific cDNA molecules present in the samples was determined by absolute quantification method of qPCR analysis [45]. A range of six dilutions (10^7^–10^2 ^copies) of the cDNA was made and a gene-specific external standard curve was generated by using cDNA standards that were run simultaneously with the experimental samples. Change in target gene expression was calculated using equation 2^-ΔΔCT ^[87]. The fold change in the target gene, normalized to 18S rRNA (*Pm-18s) *and relative to the expression of control, was calculated for each sample. A gene with a relative abundance of one is equal to the abundance of 18S rRNA in the same sample. An *F*-test at a significance level of *P *< 0.05 was used to compare the ratio of the mean gene expression of desiccated samples with that of the control. To minimize mRNA quantification errors, genomic DNA contamination biases and to correct for inter-sample variations, we used 18s ribosomal RNAs (rRNAs) of *P. murrayi *as an internal control.

## Authors' contributions

BNA carried out most of the work described here including conception and design of experiments, acquisition of data, analysis and interpretation of data and drafting the manuscript. DHW and BJA contributed to sample and culture collection, conception and design of experiments, supervision of the work and critical review of the manuscript. All authors read and approved the final manuscript.

## Supplementary Material

Additional File 1 **Distribution of (a) molecular functions, (b) cellular components, and (c) biological process categories based on gene ontology for *Plectus murrayi *unique sequences**. Distribution of (a) molecular functions, (b) cellular components, and (c) biological process categories based on gene ontology for *Plectus murrayi *unique sequences. More information provided in Figure 2a, b and 2c. Note that individual GO categories can have multiple mappings. Click here for file

Additional File 2 **Analysis of mRNA copy number (×10^7^) of *Pm-hsp-90*: heat shock protein 90 and *Pm-hsp-70*: heat shock protein 70 gene in *Plectus murrayi *under desiccated and normal condition**. Analysis of mRNA copy number (×10^7^) of *Pm-hsp-90*: heat shock protein 90 and *Pm-hsp-70*: heat shock protein 70 gene in *Plectus murrayi *under desiccated and normal condition. The experiment was performed using an absolute quantitation method of quantitative real-time PCR analysis with each value represents the mean ± SE of three replicates. Nematode samples were exposed to 97 and 85% RH for 3 and 2 days respectively prior to RNA extraction. Controls received no treatment. *Significant difference (P < 0.05) from control. Click here for file

Additional File 3 **List of the gene specific primer sequences used for quantitative real-time PCR analysis**. List of the gene specific primer sequences used for quantitative real-time PCR analysis. Primers were designed by aligning the EST sequences with their putative homologue from GenBank using IDT SciTools (Integrated DNA Technologies, Coralville, IA, USA) and synthesized by Operon (Operon Biotechnologies Inc., Huntsville, AL, USA). Click here for file
