# Supplementary material for: Desiccation survival in an Antarctic nematode: molecular analysis using expressed sequenced tags
Source: BMC Genomics. 2009 Feb 9;10:69. doi: 10.1186/1471-2164-10-69 (PMC2667540; doi:10.1186/1471-2164-10-69)
Supplement: Additional File 1 — Distribution of (a) molecular functions, (b) cellular components, and (c) biological process categories based on gene ontology for Plectus murrayi unique sequences. Distribution of (a) molecular functions, (b) cellular components, and (c) biological process categories based on gene ontology for Plectus murrayi unique sequences. More information provided in Figure 2a, b and 2c. Note that individual GO categories can have multiple mappings. [file 1471-2164-10-69-S1.doc]

**Additional file 1-** Distribution of (A) molecular functions, (B) cellular components and (C) biological process based on gene ontology for *Plectus murrayi* unique sequences.

**A) Molecular Functions**

Categories and subcategories Representation % Representation^‡^

Ligand binding/carrier 232 48%

Protein binding 87 18%

Nucleotide binding 55 11%

Nucleic acid binding 49 10%

Calcium binding 26 5%

Cofactor binding 15 3%

Enzyme 138 28%

Oxidoreductase 45 9%

Hydrolase 43 9%

Transferase 23 5%

Phosphatase 15 3%

Isomerase 4 <1%

Helicase 3 <1%

Lyase 3 <1%

Ligase 2 <1%

Molecular transducer 31 6%

Receptor 17 3%

Signal transducer 14 3%

Transporter 28 6%

Substrate specific transporter 19 4%

Transmembrane transporter 9 2%

Structural molecule 20 4%

Enzyme regulator 19 4%

Translation regulation 13 3%

Transcriptional regulation 6 1%

**(B) Cellular components**

Categories and subcategories Representation % Representation^‡^

Cell 209 92%

Intracellular 176 78%

Cytoplasm 102 45%

Mitochondria 28 12%

Cytosol 25 11%

Ribosome 20 9%

Vesicle 15 7%

Endoplasmic reticulum 5 2%

Cytoskeleton 5 2%

Golgi apparatus 4 2%

Protein complex 33 15%

Nucleus 26 12%

Ribonucleoprotein complex 12 5%

Cell fraction 3 1%

Membrane 33 15%

Integral membrane 13 6%

Mitochondrial membrane 11 5%

Plasma membrane 9 4%

Extracellular 17 8%

**(C) Biological process**

Categories and subcategories Representation % Representation^‡^

Cell growth and/or maintenance 241 66%

Metabolism 145 40%

Protein metabolism 44 12%

Proteolysis 23 6%

Protein modification 13 4%

Protein folding 8 2%

Biosynthesis 19 5%

Carbohydrate metabolism 18 5%

Nucleic acid metabolism 16 4%

RNA metabolism 11 3% DNA metabolism 5 1%

Oxygen and reactive oxygen metabolism 15 4%

Catabolism 13 4%

Lipid metabolism 9 2%

Phosphate metabolism 6 2%

Amino acid and derivative metabolism 5 1%

Localization 23 6%

Cell organization and biogenesis 22 6%

Transport 21 6%

Response to stress 16 4%

Cellular respiration 6 2%

Cell cycle 5 2%

Homeostasis 3 <1%

Development 61 17%

Embryonic development 11 3%

Growth 10 3%

Post-embryonic development 10 3%

Reproduction 8 2%

Larval development 8 2%

Cell differentiation 7 2%

Transcription 7 2%

Cell communication 39 11%

Response to stimulus 26 7%

Signal transduction 13 4%

Intracellular signalling cascade 8 2%

Cell surface receptor linked 5 1%

Behavior 15 4%

Programmed cell death 8 2%

^‡^Percentage representation based on 487, 226 and 364 unique sequences for (A), (B) and (C) respectively.
