# Supplementary material for: Desiccation survival in an Antarctic nematode: molecular analysis using expressed sequenced tags
Source: BMC Genomics. 2009 Feb 9;10:69. doi: 10.1186/1471-2164-10-69 (PMC2667540; doi:10.1186/1471-2164-10-69)
Supplement: Additional File 2 — Analysis of mRNA copy number (×107) of Pm-hsp-90: heat shock protein 90 and Pm-hsp-70: heat shock protein 70 gene in Plectus murrayi under desiccated and normal condition. Analysis of mRNA copy number (×107) of Pm-hsp-90: heat shock protein 90 and Pm-hsp-70: heat shock protein 70 gene in Plectus murrayi under desiccated and normal condition. The experiment was performed using an absolute quantitation method of quantitative real-time PCR analysis with each value represents the mean ± SE of three replicates. Nematode samples were exposed to 97 and 85% RH for 3 and 2 days respectively prior to RNA extraction. Controls received no treatment. *Significant difference (P < 0.05) from control. [file 1471-2164-10-69-S2.doc]

**Additional file 2-** Analysis of mRNA copy number (×10^7^) of *Hsp90*: heat shock protein 90 and *Hsp70*: heat shock protein 70 gene in *Plectus murrayi* under desiccation and normal condition.

| Gene | Treatment | |
| --- | --- | --- |
|  | Desiccated | Control |
| Hsp90 | 6.73±0.58 | 6.45±1.1 |
| Hsp70 | 7.20±0.96 | 7.45±1.19 |
