# Supplementary material for: Desiccation survival in an Antarctic nematode: molecular analysis using expressed sequenced tags
Source: BMC Genomics. 2009 Feb 9;10:69. doi: 10.1186/1471-2164-10-69 (PMC2667540; doi:10.1186/1471-2164-10-69)
Supplement: Additional File 3 — List of the gene specific primer sequences used for quantitative real-time PCR analysis. List of the gene specific primer sequences used for quantitative real-time PCR analysis. Primers were designed by aligning the EST sequences with their putative homologue from GenBank using IDT SciTools (Integrated DNA Technologies, Coralville, IA, USA) and synthesized by Operon (Operon Biotechnologies Inc., Huntsville, AL, USA). [file 1471-2164-10-69-S3.doc]

**Additional file 3-** List of the gene-specific primers used in quantitative real-time PCR analysis.

Primer name Gene Primer forward 5’ to 3’ Primer reverse 5’ to 3’ Product length

(bp)

AlDeh Aldehyde dehydrogenase TGCTTGTGGACCAATCACGCAATC ATGGAACGTCTGCCCAATACTCGT 222
Tps Trehalose-6-phosphate synthase GCACGACAAGCAACGAGTTA CATGTTCACACCAAGGTTCG 180
GlPer Glutathione peroxidase TGCTCATCCACTGTGGCAGTTTC AGTGAGCTGGGAGATGTTGTTGGA 156

Afp Antifreeze protein GAGTTGCAAGTCCAACCCAAACCA CATTCCAAAGGGTGCCATTGTCGT 192
Hsp70 Heat shock protein 70 AGTTGGGAGCAATCATGGCCAAAG GCGACTTGATTCTTGGCAGCATCT 255

Lea Late embryogenesis abundant protein ACAACGAGGCCAAGGGAAAGACT TTTGTCGGAGGCCTTGTCCTTGTA 187

GlKin Glycerol kinase TAAGTCAGTGGGCGTGGCTAATCA CACAATTGCGTTGTAGAGCGGCTT 205

MalSyn Malate synthase CACTATCGCTCGTTCGTCAA CCGGCATCTGTTCTAGTTCC 211

GlySyn Glycogen synthase ATGAATGGCAAGCAGGTGTTGGTC AACGATCCGAGATGTGTAGTCGCA 188

Hsp90 Heat shock protein 90 TGCAAACATCTGGAAACCAA CCAAACTGGCCAATAATGCT 227

Rpl-4 Ribosomal protein TGAGGTCCCACTTGTTGTTTCCGA TAACAACTGGTCCGAGCTTCTGCT 235

Nov-76 Novel protein I CCGCTTATTGGGGTTGTCTA ATCCGTCCCTACAATCAGCAGCAT 164

Nov-80 Novel protein II ACTACTCCCGCAGAACAACCAACA CTCCCGTTGAACGAAACACT 192

Gst-1 Glutathione s-transferase 1 TGCCTATGGATTCGCTGATGGAGA AGGGAAAGCAGAGATTGCATTGGG 217
